# Mobility Control Mechanism of In Situ Viscosity-Enhancing Graphene Quantum Dots in Assisted CO_2_ Flooding

**DOI:** 10.3390/molecules31121997

**Published:** 2026-06-07

**Authors:** Fang Shi, Weibin Jin, Jingchun Wu, Bo Zhao, Chunlong Zhang, Lifeng Mao

**Affiliations:** 1 Key Laboratory for EOR Technology (Ministry of Education), Northeast Petroleum University, Daqing 163318, China; jwb090218@163.com (W.J.); w6529@163.com (J.W.); 2 Joint International Research Laboratory of Enhanced Oil & Gas Recovery, Kazakh-British Technical University, Tole Bi Street 59, Almaty 050000, Kazakhstan; 3 Daqing Oil Field Co., Ltd., No. 6 Oil Production Plant, Daqing 163000, China; 4 Daqing Yongzhu Petroleum Technology Development Co., Ltd., Daqing 163000, China

**Keywords:** shale reservoirs, graphene quantum dots, amidine functionalization, CO_2_-responsive, enhanced oil recovery

## Abstract

To address gas channeling, low sweep efficiency, and water sensitivity in CO_2_ flooding of shale reservoirs, amidine-functionalized graphene quantum dots (FN-GQDs) were synthesized via amidation of citric acid-derived GQDs. FTIR and UV-Vis confirmed successful grafting. Conductometric titration showed an optimal reaction time of 24 h with a grafting ratio of 58%, in good agreement with the 60% saturation predicted by molecular dynamics simulation. Upon CO_2_ introduction, protonation of amidine groups induced a nonlinear viscosity increase from 0.298 to 2.0 mPa·s at 0.02 wt% via electrostatic attraction and hydrogen bonding, forming a dynamic crosslinking network. FN-GQDs maintained low oil-water interfacial tension of 0.12–0.25 mN/m at 80–120 °C and rapidly reversed rock wettability from strongly oil-wet to water-wet, reducing the contact angle from 141.7° to 38.9° within 80 min. The positively charged surface inhibited clay swelling, achieving 92% at 0.20 wt%. Core flooding and NMR T_2_ spectra revealed that alternating CO_2_ and FN-GQDs injection at a 2:1 gas–water ratio achieved a final oil recovery of 52.5%, significantly higher than pure CO_2_ flooding. Through synergistic effects of interfacial tension reduction, wettability alteration, viscosity enhancement, and anti-swelling, FN-GQDs improve microscopic displacement efficiency and macroscopic sweep volume, showing great potential for CO_2_-enhanced oil recovery in shale reservoirs.

## 1. Introduction

As the degree of petroleum resource exploitation increases year by year, the targets of oil and gas development are gradually shifting toward unconventional reservoirs, such as low-permeability, ultra-low-permeability, and shale oil reservoirs. Against the backdrop of the “dual-carbon” strategy and the rapid advancement of CCUS technology [1,2]. CO_2_ flooding has emerged as one of the important methods for enhancing oil recovery in shale reservoirs [3,4]. As a small gas molecule, CO_2_ exhibits favorable mobility in nanoscale pores [5]. It is capable of dissolving in crude oil and reducing its viscosity [6]. Under suitable conditions, miscible displacement can be achieved [7]. In theory, a high oil displacement efficiency is achievable [8]. However, in practical field applications, due to the significant mobility ratio disparity between CO_2_ and crude oil, severe gas channeling is commonly encountered during CO_2_ flooding [9]. This results in an unstable displacement front and a low sweep volume, and a final oil recovery that is far below the theoretical expectation [10]. Therefore, how to effectively suppress CO_2_ channeling and enlarge the sweep volume has become a key issue to address in the application of CO_2_ flooding. To mitigate gas channeling, water-alternating-gas (WAG) injection is commonly employed in field practices both domestically and internationally. By using water slugs to block high-permeability channels, WAG forces subsequent CO_2_ to divert, thereby enlarging the sweep volume. In the Weyburn oilfield in Canada and the SACROC oilfield in the United States, the application of WAG technology has led to an improvement in oil recovery during CO_2_ flooding [11]. However, in shale reservoirs, the proportion of montmorillonite clay minerals is relatively high, which makes them highly susceptible to hydration swelling upon contact with water [12]. This leads to pore throat blockage and a sharp decline in permeability, resulting in severe reservoir damage [13,14]. A study by He Jingang et al. on shale reservoirs showed that due to the abundance of clay minerals, shale reservoirs generally suffer from moderate to strong water-sensitive damage. It was found that fluid damage increased the stress sensitivity coefficient by an average of 30%, shifting the degree of damage from moderate to strong stress sensitivity [15].

Therefore, there is an urgent need to find a novel displacement medium that can replace the water phase and effectively control CO_2_ mobility. To address the above issues, stimuli-responsive materials can be employed for mobility control in CO_2_ flooding. Among them, CO_2_-stimuli-responsive materials offer advantages such as low cost and easy availability of the stimulus [16], a clean and reversible response process, and no residual contamination [17,18]. They are well-suited for CO_2_ flooding scenarios. Common CO_2_-responsive functional groups include primary amines [19], tertiary amines, amidines, and guanidines [20,21]. The study by Zhang [22] showed that compared with traditional amine functional groups, amidine groups exhibit a stronger CO_2_ binding affinity. Protonation can occur rapidly at lower CO_2_ concentrations, forming amidinium cation and bicarbonate ion pairs [23]. On a macroscopic scale, the amidine-containing solution exhibits an increase in viscosity upon CO_2_ stimulation [24]. This characteristic can be utilized to construct a CO_2_ “in situ-activated” oil displacement system. In terms of nanoparticle selection, graphene quantum dots (GQDs) are characterized by their unique zero-dimensional structure [25,26], ease of surface modification [27], favorable water dispersibility, and environmentally friendly properties [28]. In recent years, they have garnered widespread attention in the field of oil and gas field development [29]. With a size of less than 10 nm, GQDs can effectively enter the nanoscale pore throats of shale reservoirs and exert interfacial regulation effects under nanoconfinement [30]. The study by Yin et al. [31] demonstrated that functionalized GQDs can reduce the oil–water interfacial tension and alter rock wettability even at low concentrations. This offers the advantage of “low concentration, high efficiency.“ Their carbon-based backbone is free from secondary pollution in reservoir environments, exhibiting environmentally friendly characteristics. Based on the above analysis, this study proposes covalently grafting amidine functional groups onto the surface of graphene quantum dots. CO_2_-stimuli-responsive graphene quantum dots (FN-GQDs) were constructed. During the application of this material, CO_2_ serves as the trigger, enabling in situ activation at depth within the reservoir. In the absence of CO_2_, the amidine groups exist in a neutral form, and FN-GQDs maintain a small hydrodynamic radius and low viscosity. This facilitates injection and transport within the micro-/nano-pores of shale reservoirs. Upon contact with CO_2_, rapid protonation of the amidine groups occurs. Positively charged cationic groups are formed, which electrostatically adsorb onto the negatively charged rock surface to form a film. Wettability reversal is achieved, and clay swelling is effectively inhibited, thereby resolving the issue of water-sensitive damage. Additionally, the protonated FN-GQDs form a dynamic crosslinking network through the synergistic effects of electrostatic attraction and hydrogen bonding. The solution viscosity is significantly increased, thereby improving the mobility ratio between CO_2_ and crude oil, suppressing gas channeling, and enlarging the sweep volume. Targeted control during the CO_2_ flooding process is thereby achieved.

In this study, GQDs with carboxyl-rich surfaces were first synthesized via a hydrothermal method. Subsequently, amidine functional groups were grafted via an amidization reaction, yielding the target product FN-GQDs. The material structure was characterized using FTIR, UV-Vis, particle size analysis, and other techniques. The grafting ratio and reaction time were optimized through conductometric titration and molecular dynamics simulation. The viscosity variation, interfacial tension, wettability reversal, and anti-swelling performance of FN-GQDs before and after CO_2_ stimulation were systematically evaluated. Finally, through core flooding experiments and nuclear magnetic resonance (NMR) technology, it was revealed that FN-GQDs, during CO_2_ flooding, mitigate reservoir damage by inhibiting clay swelling and regulate the mobility ratio by increasing viscosity upon CO_2_ stimulation. Therefore, enhanced oil recovery was achieved in an auxiliary manner. This study aims to provide a new synthesis method and theoretical basis for nanomaterials used in synergistic CO_2_ flooding.

## 2. Results

### 2.1. Structural Characterization of FN-GQDs

**Figure 1:**
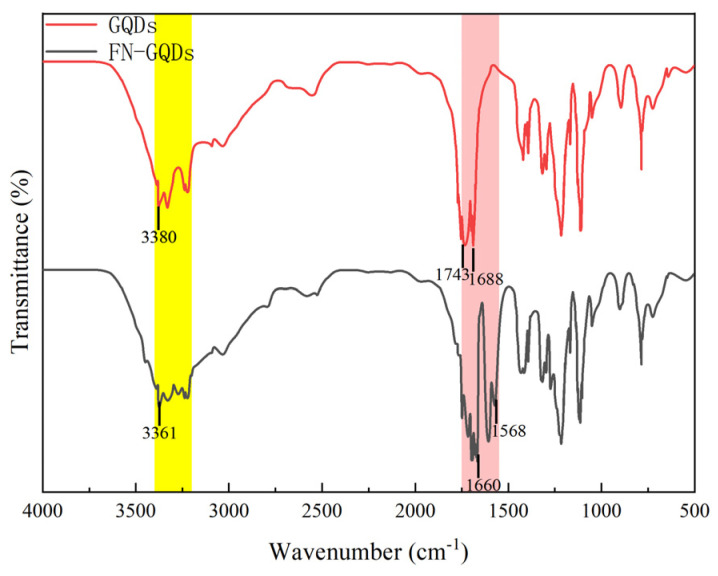
FTIR spectra of GQDs and FN-GQDs.

Figure 1 shows the FTIR spectra of GQDs and FN-GQDs. From the red curve in Figure 1, a broad and intense absorption peak was observed for GQDs at 3380 cm^−1^. This was attributed to the stretching vibration of -OH bonds in carboxyl and hydroxyl groups. An absorption peak of moderate intensity was observed near 1743 cm^−1^, corresponding to the stretching vibration of C=O in carboxyl groups. The absorption peak at 1688 cm^−1^ was assigned to the stretching vibration of the aromatic ring skeleton (C=C). These characteristic features were in good agreement with the infrared characteristics of graphene quantum dots prepared by citric acid pyrolysis, which are known to be rich in carboxyl and hydroxyl groups on their surfaces. This indicates that the synthesized GQDs were consistent with the theoretical molecular structure.

As shown in Figure 1, the characteristic absorption peak of carboxyl C=O at 1743 cm^−1^ was significantly weakened in intensity. Meanwhile, two new absorption peaks appeared at 1660 cm^−1^ and 1568 cm^−1^. The absorption peak at 1660 cm^−1^ was attributed to the stretching vibration of C=O in the amide bond. The absorption peak at 1568 cm^−1^ was assigned to the bending vibration of N-H and the stretching vibration of C-N in the amide bond. In addition, due to the superimposed absorption of the introduced amide bonds and the N-H bonds in the amidine groups, the O-H/N-H stretching vibration peak in the range of 3400–3300 cm^−1^ broadened. The above results indicate that after activation by EDC/NHS, the carboxyl groups on the surface of GQDs successfully underwent amidization with aminoethylamidine. Amide bonds were formed, and amidine functional groups were introduced. This indicates that the synthesized FN-GQDs were consistent with the theoretical molecular structure.

**Figure 2:**
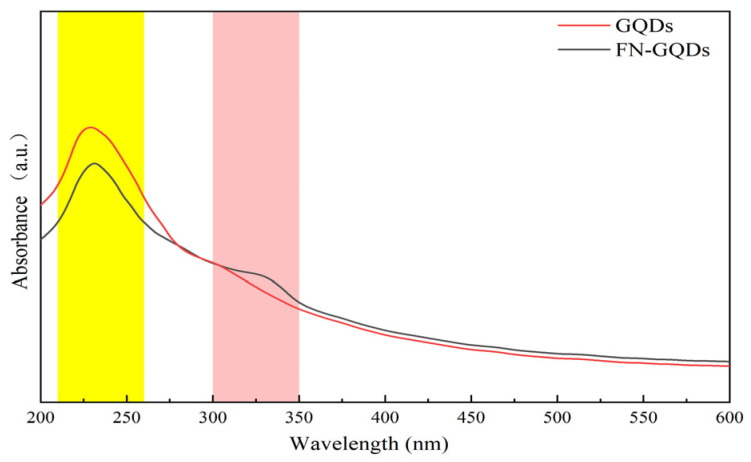
UV-Vis spectra of GQDs and FN-GQDs.

The UV-Vis spectra of GQDs and FN-GQDs are presented in Figure 2. It can be seen in Figure 2 that a strong and sharp absorption peak was exhibited by GQDs at 225 nm, which is a typical feature caused by the π → π* electronic transition of the aromatic sp^2^ domains in the carbon core of the graphene quantum dots. After this strong absorption peak, the absorbance decreased sharply with the increase in wavelength, and only a very low background absorption was maintained in the long-wavelength region beyond 300 nm, corresponding to the n → π transition of the surface oxygen-containing functional groups. Compared with GQDs, the characteristic π → π* absorption peak of FN-GQDs was red-shifted to 230 nm, and its intensity was slightly reduced. A new absorption shoulder peak was observed for FN-GQDs at around 320 nm, and the background absorption in the region beyond 400 nm was slightly enhanced. The above changes are attributed to the fact that amide bonds and amidine functional groups were introduced through the amidation of carboxyl groups on the surface of the GQDs. Additional lone-pair electrons were provided by these nitrogen-containing groups, the contribution of the n → π* transition was enhanced, and the redistribution of the electronic energy levels was caused by the reconstruction of the surface states after functionalization. Consequently, the absorption peak of the GQDs around 300 nm exhibited broadening and a red shift. It was demonstrated by the red shift and broadening of the characteristic absorption peaks of FN-GQDs that aminoethylamidine had been successfully grafted onto the GQDs surface via covalent amide bonds, and the formed amidine-functionalized graphene quantum dots were consistent with the designed molecule.

### 2.2. Optimization of Grafting Ratio

**Figure 3:**
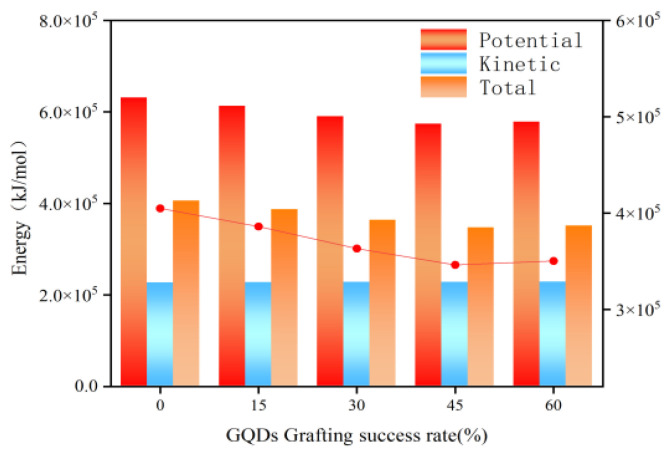
Evolution of potential, kinetic, and total energy of FN-GQDs over a 20 ns MD simulation (20 ns).

The temporal evolution profiles of the total energy, kinetic energy, and potential energy of the FN-GQDs systems at grafting ratios of 0%, 15%, 30%, 45%, and 60% are presented in Figure 3. The solid lines represent the running averages of the corresponding energy components. The left y-axis represents the potential energy and total energy, while the right y-axis represents the kinetic energy.

All energy terms calculated from the simulation were found to be negative. As the grafting ratio was increased from 0% to 45%, the absolute value of the total energy was observed to decrease from 404.95 × 10^3^ kJ/mol to 346.25 × 10^3^ kJ/mol. When the grafting ratio was further raised to 60%, the total energy was slightly recovered to 350.35 × 10^3^ kJ/mol. The decrease in the absolute value of the total energy indicates that the system was evolving toward a less negative, higher-energy state. This trend suggests that after the introduction of amidine chains onto the GQD surface, the steric repulsion and conformational strain among the grafted chains increased, partially offsetting the stabilization conferred by covalent attachment. Nevertheless, the difference in total energy between the 45% and 60% grafting ratios was merely 4.10 × 10^3^ kJ/mol, corresponding to a variation of only about 1%, which is of the same order of magnitude as the thermal fluctuation at the simulation temperature. Therefore, the systems with 45% and 60% grafting ratios could be regarded as isoenergetic, and the grafting-driven energy change was essentially saturated at this level. The topological structures of the products at each grafting condition are displayed in Table 1. Further molecular dynamics simulations confirmed that when the grafting ratio exceeded 60%, the simulation terminated with an error and no result could be displayed. Hence, the energy data indicate that a maximum amidine grafting ratio of approximately 60% could be accommodated by the GQDs without compromising their structural integrity. The saturation grafting ratio predicted by the simulation is in good agreement with the value of 58% subsequently obtained from conductometric titration experiments.

Through simulation, it was found that when the grafting ratio of functional groups on the graphene quantum dots exceeded 60%, deformation of the GQDs structure occurred. The mechanical parameters decreased, and the molecular interactions became imbalanced, resulting in entanglement and agglomeration of the molecular chains. Therefore, the grafting ratios were set to 0%, 15%, 30%, 45%, and 60%. The top and side views of the simulated topological structures of the graphene quantum dots with the five grafting ratios are shown in Table 1.

### 2.3. Optimization of Reaction Time

**Figure 4:**
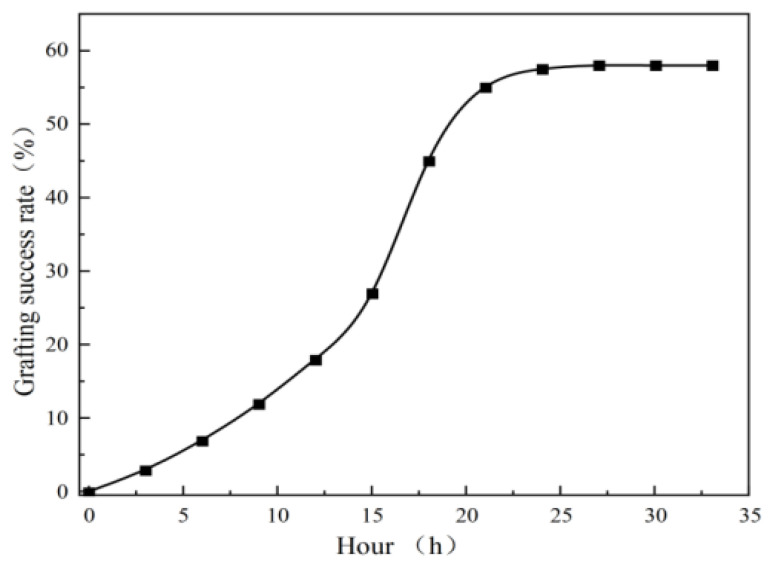
Relationship between the grafting ratio of amidine groups on FN-GQDs and reaction time.

Conductometric titration determined the grafting ratios of amidine groups on GQDs at different reaction times, as shown in Figure 4. The grafting ratio increased rapidly initially and then leveled off. At 6 h, it was 7%; at 9 h, 12%; at 18 h, it reached 45%. The fast initial rate was due to abundant activated carboxyl sites on GQDs, allowing rapid amidization with aminoethylamidine. At 21 h, the ratio reached 55%. Extending the reaction to 33 h, the ratio remained around 58%, indicating equilibrium after about 24 h with a maximum grafting ratio of approximately 58%.

Combining titration with molecular dynamics simulation, the experimental plateau of 58% agreed well with the simulated saturation grafting ratio of 60%. Two factors explain this plateau. First, some carboxyl groups on the GQDs surface are sterically hindered or embedded within sheet layers, preventing effective contact with aminoethylamidine. Second, simulations confirmed that a grafting ratio exceeding 60% would compromise the structural stability of GQDs, leading to molecular chain entanglement and agglomeration. Therefore, during actual synthesis, the grafting ratio saturates at about 58%.

In summary, the conductometric titration results indicate that aminoethylamidine was successfully grafted onto the surface of GQDs. The optimal reaction time was determined to be 24 h, at which point the grafting ratio reached a plateau value of 58%.

### 2.4. Morphology Structure and Particle Size Analysis

**Figure 5:**
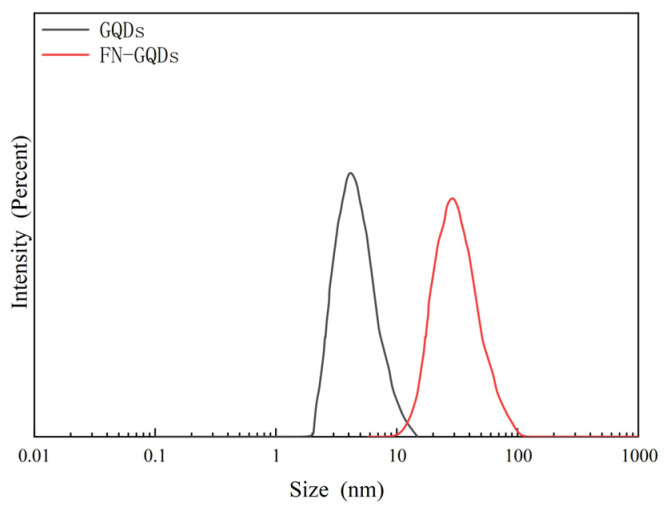
Particle size distribution of unmodified GQDs and modified FN-GQDs.

The particle size distributions of GQDs and FN-GQDs were determined using a nanoparticle size analyzer. The results are shown in Figure 5. The median particle size of the unmodified GQDs was 4.2 nm. After amidine modification, the median particle size of FN-GQDs was 26.1 nm. A significant increase in particle size was observed after functionalization. From the particle size distribution graph, it can be seen that the particle size distribution of GQDs was relatively narrow. The distribution was mainly in the range of 3–6 nm. This indicates that the graphene quantum dots prepared by the citric acid pyrolysis method exhibited good size uniformity. After amidine functionalization, the particle size distribution of FN-GQDs shifted toward larger dimensions. Aminoethylamidine molecules were covalently grafted onto the surface of GQDs via amide bonds. The grafted functional groups increased the hydrodynamic radius of the quantum dots. The increase in particle size was consistent with the results of FTIR and UV-Vis analyses. This further confirms that amidine functional groups were successfully grafted onto the surface of GQDs.

### 2.5. CO_2_ Stimulus-Response Testing

**Figure 6:**
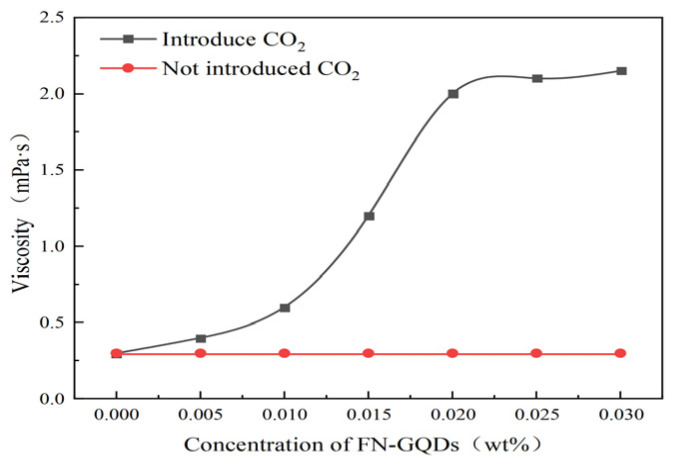
Viscosity of FN-GQDs solution as a function of concentration before and after CO_2_.

As shown in Figure 6, the viscosity of the FN-GQDs solution after CO_2_ introduction increased nonlinearly with the concentration of FN-GQDs. At 0 wt%, viscosity was 0.298 mPa·s. At 0.01 wt%, it reached 0.6 mPa·s, indicating that at very low concentrations, interparticle spacing was large and protonation-induced electrostatic attraction and hydrogen bonding were insufficient to form a crosslinking network, keeping viscosity low. At 0.02 wt%, viscosity sharply rose to 2.0 mPa·s, demonstrating that positively charged amidine cations began forming a dynamic crosslinking network via electrostatic attraction and hydrogen bonding, increasing hydrodynamic volume and macroscopic viscosity. Beyond 0.02 wt%, the network approached saturation, and further concentration increase did not significantly affect viscosity. In the absence of CO_2_, amidine groups remained neutral, and quantum dots interacted only through van der Waals forces, showing no notable viscosity change. Upon CO_2_ introduction, protonation generated positively charged amidine cations and bicarbonate ion pairs, enabling network formation and viscosity increase. Based on these results, an FN-GQDs concentration of 0.02 wt% was selected for subsequent experiments.

### 2.6. Analysis of Anti-Swelling Performance

**Figure 7:**
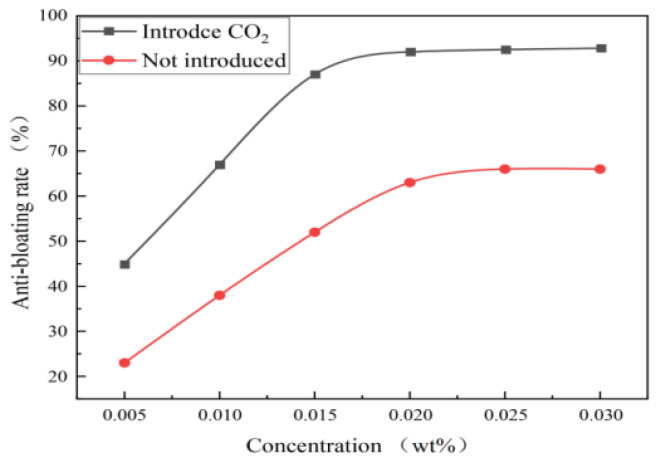
Anti-swelling rate of FN-GQDs solution as a function of concentration.

After CO_2_ introduction, FN-GQDs exhibited strong anti-swelling performance. As shown in Figure 7, the anti-swelling rate increased with increasing concentration. At a concentration of 0.15 wt%, the anti-swelling rate reached 87%. Saturation was reached at 0.02 wt%, after which the anti-swelling rate was 92%. Under CO_2_ stimulation, the surface of FN-GQDs became positively charged. Adsorption occurred on the surface of negatively charged clay sheets. Surface charges were neutralized, and the electrostatic repulsion between clay layers was weakened. Thus, interlayer expansion was inhibited. After protonation, a dense adsorption film was formed by the material on the clay surface. As a result, clay water-sensitive swelling in shale reservoirs was effectively inhibited. The addition of FN-GQDs was shown to reduce reservoir damage during the water-alternating-gas process. Therefore, an FN-GQDs solution with a concentration greater than 0.15 wt% was selected for application. By comprehensively considering the experimental results from Section 2.4, a concentration of 0.02 wt% FN-GQDs solution was selected for subsequent experiments.

### 2.7. Oil–Water Interfacial Tension

**Figure 8:**
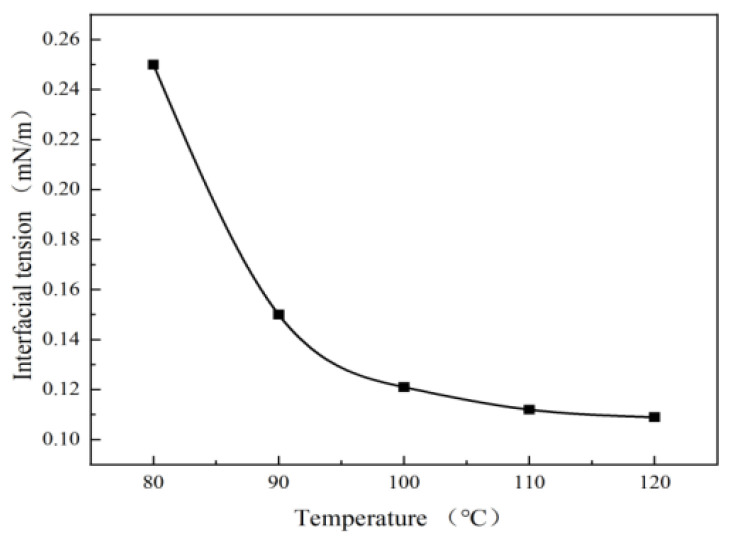
Oil–water interfacial tension of FN-GQDs solution as a function of temperature.

As shown in Figure 8, the interfacial tension of the FN-GQDs solution exhibited a decreasing trend with increasing temperature. At 80 °C, the interfacial tension was 0.25 mN/m. When the temperature increased to 100 °C, it rapidly decreased to 0.12 mN/m. After 100 °C, the interfacial tension decreased slowly with further increases in temperature. Under high-temperature conditions of 80–100 °C, FN-GQDs were still able to maintain a low interfacial tension. This indicates that the FN-GQDs solution exhibited good thermal stability and oil–water interfacial activity. The low interfacial tension made it easier for oil droplets to be stretched and broken into oil filaments during flow. Thereby, microscopic oil displacement efficiency was enhanced.

### 2.8. Rock Wettability

**Figure 9:**
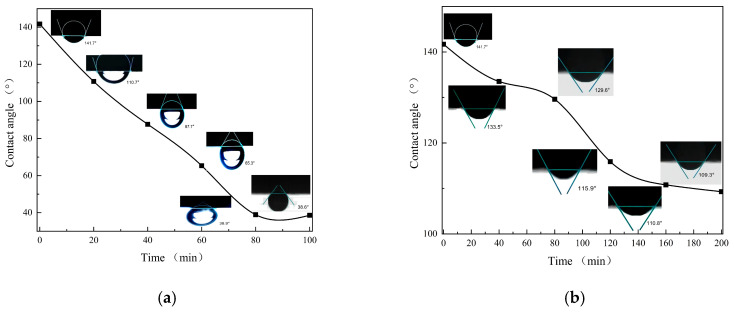
(**a**) Variation in rock surface contact angle over time after CO_2_ introduction. (**b**) Variation in rock surface contact angle over time without CO_2_ introduction.

Figure 9a shows that after CO_2_ introduction, the rock contact angle in FN-GQDs solution decreased over time, indicating a gradual transition from oil-wet to water-wet. and finally. to a superhydrophilic state. Without quantum dots, the contact angle was 141.7°. After 80 min, it rapidly dropped to 38.9° and then stabilized, demonstrating rapid wettability reversal. Figure 9b shows that without CO_2_, the contact angle remained above 90° throughout, and the rock stayed oil-wet, stabilizing at 109.3° after 200 min. At the same time point, the contact angle with CO_2_ was significantly lower. This rapid reversal is attributed to CO_2_-induced protonation of amidine groups on FN-GQDs, forming positively charged cations that quickly adsorb onto the rock surface. Through electrostatic interactions and hydrogen bonding, FN-GQDs form a dense adsorption layer, and their amphiphilic structure arranges in an ordered manner at the interface, effectively shielding original oil-wet groups and allowing water to spread fully. Over time, the adsorption layer becomes more ordered and denser, continuously decreasing the contact angle until a superhydrophilic state is reached. In the absence of CO_2_, amidine groups remain neutral, resulting in weak interfacial activity and a limited effect on wettability.

To quantitatively evaluate the dynamic wettability alteration rate, the contact angle data in Figure 9 were analyzed using a linear kinetic model. In the presence of CO_2_, the contact angle decreased linearly over time during the initial rapid-change stage from 0 to 80 min, yielding a rate constant k = 1.255°/min with an excellent linear correlation (R^2^ = 0.997). This linearity indicates that the CO_2_-driven protonation and subsequent adsorption of FN-GQDs onto the rock surface followed a pseudo-zero-order process within this period. In sharp contrast, in the absence of CO_2_ (Figure 9b), the contact angle diminished much more slowly over the entire 200 min observation, giving a rate constant of only 0.1745°/min (R^2^ = 0.955). The approximately 7.2-fold enhancement in the wettability reversal rate upon CO_2_ exposure quantitatively confirms the rapid CO_2_-responsive interfacial activity of FN-GQDs.

### 2.9. Analysis of Synergistic Enhanced Oil Recovery Mechanism

**Figure 10:**
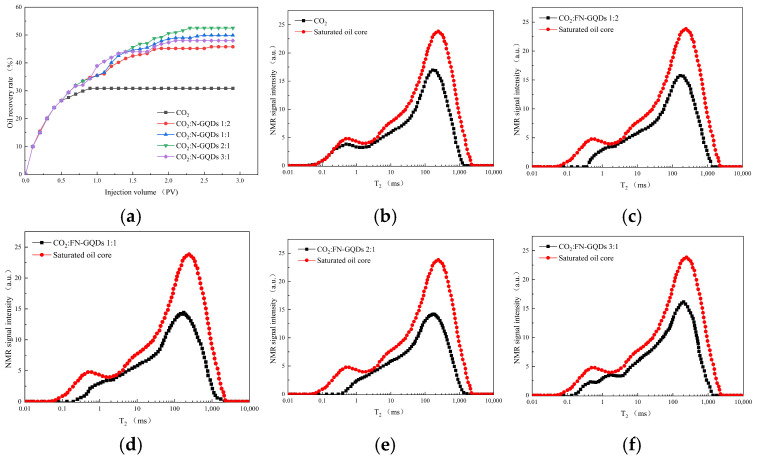
(**a**) Relationship between injection volume and oil displacement efficiency. (**b**) NMR T_2_ spectra under CO_2_ flooding, (**c**) NMR T_2_ spectra at a gas–water ratio of 1:2, (**d**) NMR T_2_ spectra at a gas–water ratio of 1:1, (**e**) NMR T_2_ spectra at a gas–water ratio of 2:2, (**f**) NMR T_2_ spectra at a gas–water ratio of 3:1.

The variation in oil recovery with injection volume under different displacement media and different gas–water ratios was evaluated using a core flooding experimental setup. The results are shown in Figure 10a. The flooding methods included pure CO_2_ flooding and CO_2_ and FN-GQDs alternating flooding (gas–FN-GQDs alternating). The different CO_2_:FN-GQDs gas–water ratios included 3:1, 2:1, 1:1, and 1:2. Based on the preliminary performance evaluation experiments, an FN-GQDs concentration of 0.02 wt% was selected for the flooding experiments. For both flooding methods, the oil recovery increased with increasing injection volume. After the injection volume reached 2.5 PV, the oil recovery stabilized. Under all gas–water ratios, the oil recovery was significantly higher than that of pure CO_2_ flooding. Under all gas–water alternating injection schemes, the injection pressure was consistently maintained stable, no sudden pressure jump or loss of injectivity was observed, confirming that pore throat plugging was not caused by CO_2_ in situ viscosification, and that the FN-GQDs system exhibited good injectivity under reservoir conditions. When the optimal gas–water ratio was 2:1, the oil recovery reached 52.5%. When the gas–water ratio increased to 3:1, gas channeling occurred, and the oil recovery decreased to 48.1%. Therefore, in practical applications, a gas–water ratio of 2:1 can be used.

Based on the transverse relaxation time (T_2_), the core pores can be classified into small pores (T_2_ relaxation time < 10 ms), medium pores (T_2_ relaxation time between 10 ms and 100 ms), and large pores (T_2_ relaxation time > 100 ms). Figure 10b shows the NMR T_2_ spectra before and after CO_2_ flooding. As can be seen in the figure, CO_2_ flooding primarily mobilized crude oil in medium and large pores, while the mobilization of crude oil in small pores was insufficient. This is because after CO_2_ and crude oil become miscible, the oil–gas interfacial tension decreases sharply. As a result, the fingering phenomenon is intensified. CO_2_ tends to break through more easily in the core. After breakthrough, CO_2_ flows along the preferential pathways. Thus, it is unable to fully contact the crude oil in the micro-/nano-pores. Figure 10c–f show the NMR T_2_ spectra under different gas–water ratios. Compared with CO_2_ flooding, the mobilization efficiency of small pores under gas–water alternating injection was significantly higher. In medium and large pores, the mobilization efficiency was comparable to that of CO_2_ flooding. This finding indicates that the improvement in oil recovery by gas–water alternating flooding mainly originates from the contribution of small pores. Under gas–water alternating injection, the nanoscale size of FN-GQDs in the aqueous phase enables them to enter micro-/nano-pores. As a result, efficient mobilization of crude oil in micro-/nano-pores is achieved through the reduction of interfacial tension and the alteration of rock surface wettability. Additionally, upon contact with CO_2_, in situ viscosity enhancement of the FN-GQDs solution can be achieved at depth within the reservoir. This reduces the mobility ratio during CO_2_ migration in the reservoir and enlarges the sweep volume in the CO_2_ flooding process. Therefore, while maintaining the advantages of CO_2_ miscible displacement, the displacement efficiency and sweep volume are maximized.

## 3. Discussion

In this study, a CO_2_-responsive amidine-functionalized graphene quantum dot (FN-GQDs) was successfully synthesized via amidation of citric acid-derived GQDs. FTIR, UV-Vis, and particle size analysis confirmed the covalent grafting of amidine groups, with a median size increase from 4.2 nm to 26.1 nm. Conductometric titration gave an optimal reaction time of 24 h and a grafting ratio of 58%, in excellent agreement with the 60% saturation predicted by GROMACS molecular dynamics simulation. Upon CO_2_ introduction, protonation of the amidine groups triggered a nonlinear viscosity increase from 0.298 mPa·s to 2.0 mPa·s at 0.02 wt%, driven by electrostatic attraction and hydrogen bonding that formed a dynamic crosslinking network. FN-GQDs also maintained ultra-low oil–water interfacial tension (0.12–0.25 mN/m) at 80–120 °C, rapidly reversed rock wettability from strongly oil-wet to water-wet (the contact angle decreased from 141.7° to 38.9° within 80 min), and exhibited excellent anti-swelling performance (92% at 0.20 wt%) by adsorbing onto negatively charged clay surfaces. Core flooding combined with NMR T_2_ spectra demonstrated that alternating CO_2_ and FN-GQDs injection at a 2:1 gas–water ratio achieved a final oil recovery of 52.5%, which is 21.6 percentage points higher than that of pure CO_2_ flooding. The NMR analysis revealed that pure CO_2_ flooding mainly mobilized oil in medium and large pores, whereas FN-GQDs entered small pores and effectively displaced residual oil through interfacial tension reduction and wettability alteration. The in situ viscosity increase improved the mobility ratio, suppressed gas channeling, and enlarged the sweep volume. Due to their carbon-based backbone and lack of heavy metals, FN-GQDs are expected to have low environmental toxicity, which further supports their applicability in oil reservoirs.

Compared with traditional CO_2_-responsive polymers and nanoparticles, FN-GQDs are characterized by a smaller molecular size, and while exhibiting good injectivity, they can be transported over long distances in shale reservoirs. As a single nanoscale entity, FN-GQDs integrate three functions: CO_2_-triggered viscosification, interfacial tension reduction, and wettability alteration; furthermore, they also possess anti-swelling capability. The developed FN-GQDs exhibit great potential as an auxiliary agent for CO_2_ flooding in shale reservoirs, offering a new material and theoretical basis for enhanced oil recovery under CCUS technology.

## 4. Materials and Methods

### 4.1. Materials and Experimental Equipment

Natural shale cores were selected from the Songliao Basin (2.5 cm in diameter, 4 cm in length). The gas permeability was measured to be 0.2 × 10^−3^ μm^2^. Simulated formation water (salinity 14,052 mg/L, pH 7.4, NaHCO_3_ type) was used. The specific composition is shown in Table 2. Crude oil was obtained from the Songliao Basin. The reservoir temperature was 102.2 °C, and the oil viscosity under reservoir conditions was 0.121 mPa·s. The minimum miscibility pressure with CO_2_ was 29.71 MPa, and the average reservoir pressure was 35.7 MPa.

Citric acid (anhydrous grade), 1-ethyl-3-(3-dimethylaminopropyl)carbodiimide hydrochloride (EDC·HCl), N-hydroxysuccinimide (NHS), and aminoethylamidine dihydrobromide (≥95%) were all purchased from Shanghai Aladdin Biochemical Technology Co., Ltd., Shanghai, China.

The following instruments were used: Fourier transform infrared spectrometer (Nicolet iS50, Thermo Fisher Scientific, Madison, USA); laser particle size analyzer (Malvern Zetasizer Nano ZS90, Malvern Panalytical, Malvern, UK); field-emission environmental scanning electron microscope (Quanta 450, FEI Company, Hillsboro, USA); ultraviolet-visible (UV-Vis) absorption spectrometer (U-3010, Hitachi High-Technologies Corporation, Tokyo, Japan); contact angle goniometer (OCA200, DataPhysics Instruments GmbH, Filderstadt, Germany); interfacial tensiometer (SVT15N, DataPhysics Instruments GmbH, Filderstadt, Germany); micro- and nano-scale visual physical simulation system (HBWG-70, Yangzhou Huabao Petroleum Instrument Co., Ltd., Yangzhou, China); high-temperature and high-pressure reactor (PS-100ML, Yangzhou Huabao Petroleum Instrument Co., Ltd., Yangzhou, China); physical flooding simulation system (HD-B/RT-120, Yangzhou Huabao Petroleum Instrument Co., Ltd., Yangzhou, China); high-temperature and high-pressure falling-ball viscometer (LDX-SZS-SY1 B15, Yangzhou Huabao Petroleum Instrument Co., Ltd., Yangzhou, China).

### 4.2. Preparation of FN-GQDs

First, 2.0 g of citric acid was added to 50 mL of deionized water and stirred until dissolved. The solution was transferred into a 100 mL polytetrafluoroethylene (PTFE)-lined autoclave. The reaction was carried out at 200 °C for 6 h. After natural cooling, a brownish-yellow solution was obtained. Dialysis was performed using a dialysis bag with a molecular weight cutoff of 3000 Da for 48 h to remove unreacted citric acid. After freeze-drying, yellow graphene quantum dots (GQDs) were obtained for further use, with a yield of 79.3%.

**Figure 11:**
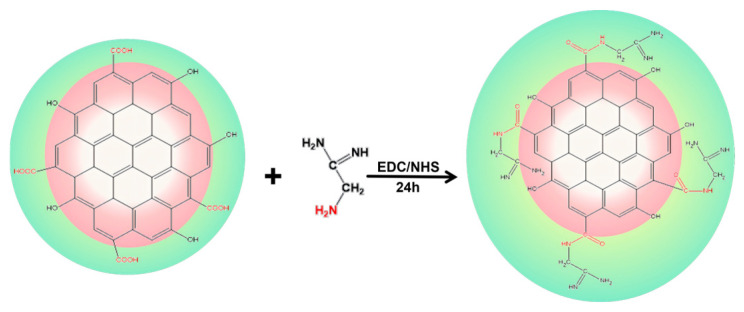
Schematic diagram of the preparation reaction process of FN-GQDs.

Then, The synthetic route is shown in Figure 11. 10 mg of graphene quantum dots was dispersed in 30 mL of PBS buffer. Ultrasonication was applied to achieve uniform dispersion, and 60 mg of EDC·HCl and 36 mg of NHS were added sequentially. The mixture was stirred in the dark at room temperature for 2 h to activate the reaction. Subsequently, 240 mg of aminoethylamidine dihydrobromide was added to the reaction system. The pH of the solution was adjusted to 7.5–8.0. The reaction was allowed to proceed for an additional 24 h at room temperature in the dark. Amidization grafting was completed, and a grafting ratio of 58% was achieved. The reaction solution was transferred entirely into a dialysis bag with a molecular weight cutoff of 10,000 Da. Dialysis was carried out at 4 °C using ultrapure water for 4 days. During this period, the water was replaced regularly to completely remove unreacted raw materials and by-products. The purified solution obtained from the dialysis bag was freeze-dried. A solid amidine-functionalized graphene quantum dots (FN-GQDs) product was obtained.

### 4.3. Structural Characterization

The structure of the amidine-functionalized graphene quantum dots was characterized using Fourier transform infrared spectroscopy. The infrared spectrometer was primarily used to characterize the molecular structure and to compare the changes in molecular bonds before and after the reaction. The scanning range was 400–4000 cm^−1^, with 32 scans and a resolution of 4. In this study, the KBr pellet method was used for sample testing.

The optical properties and structural evolution of the amidine-functionalized graphene quantum dots were characterized using a UV-Vis absorption spectrometer. Under UV-Vis irradiation, electrons in the sample molecules undergo energy level transitions, producing characteristic absorption spectra. The scanning range was 200–800 nm, with a scanning speed of 400 nm/min and a slit width set to 2 nm. In this study, the liquid sampling method was employed, and the uniformly dispersed amidine-functionalized graphene quantum dots solution was injected into a quartz cuvette. The corresponding solvent was used as a reference, and the absorbance measurement was performed.

The colloidal dispersibility and particle size distribution of the amidine-functionalized graphene quantum dots were characterized using a laser particle size analyzer. Based on the principle of light scattering, the instrument calculated the particle size distribution, average particle size, and polydispersity index of the sample. The measurement range of the instrument was 3.8 nm to 100 μm. Deionized water was used as the dispersion medium. Before measurement, the amidine-functionalized graphene quantum dots solution was diluted to an appropriate concentration. This was done to avoid interference from particle aggregation on the measurement results. Finally, the particle size distribution curve was obtained to evaluate the dispersion stability of the product.

### 4.4. Performance Evaluation

(1) Molecular dynamics simulations were performed using GROMACS(2018.8) software. The oil phase consisted of a 1:1 molar ratio of toluene to octane, and single molecules were described using the GROMACS 54A7 all-atom force field. The graphene quantum dot (GQD) was constructed as a monolayer hexagonal structure with a side length of approximately 6 nm and containing 180 edge atoms. The SPC model was used for water molecules, and periodic boundary conditions were applied in all three dimensions. The initial configuration was subjected to energy minimization using the steepest descent method to eliminate local stresses.

The simulation was conducted under the NPT ensemble for 20 ns, with a system size of 6 nm × 6 nm × 18 nm, a pressure of 35.7 MPa, and a temperature of 102.2 °C. Pressure coupling was implemented using the semi-isotropic C-rescale algorithm, with constraints applied in the x-y plane and the z-axis left open. Temperature control was achieved using a modified Berendsen thermostat. Bond lengths were constrained using the LINCS algorithm, with a time step of 1 fs. Long-range electrostatic interactions were calculated using the PME method, with a real-space cutoff of 1.2 nm. Van der Waals interactions were handled using a switching function with the same cutoff. Trajectories were collected every 10.0 ps, and periodic boundary conditions were applied in all three dimensions. The trajectories were analyzed using visualization software.

(2) The grafting ratio was controlled as follows: 100 mg of unmodified GQDs powder, dried to constant weight, was recorded as m_0_ and placed in a 50 mL beaker. Deionized water was added, and the mixture was ultrasonically dispersed to form a uniform suspension. The conductivity electrode was inserted into the suspension, and magnetic stirring was started. The initial conductivity value was recorded. Then, 0.1 mL of NaOH standard solution (0.01–0.02 mol/L) was added stepwise using a pipette. After each addition, the solution was stirred for 30 s, and once the conductivity reading stabilized, the volume V and conductivity K were recorded. Titration was continued until a clear inflection point appeared in the conductivity curve, after which an additional 3–4 data points were collected. A titration curve was plotted with NaOH volume (mL) as the horizontal axis and conductivity (μS/cm) as the vertical axis. The volume corresponding to the intersection point of the two straight lines was taken as the endpoint volume V_0_. The carboxyl content before modification was calculated using the following formula:

(1) $${\left[\mathrm{COOH}\right]}_{0}\;\left(\mathrm{mmol}/g\right)\;=\;\frac{C_{\mathrm{NaOH}}\;\times \;V_{0}\;\left(\mathrm{mL}\right)}{m_{0}\;\left(g\right)}$$

Formula: $C_{\mathrm{NaOH}}$—concentration, mol/L; $V_{0}$—endpoint volume for the unmodified sample, mL; $m_{0}$—mass of the unmodified powder, g.

The dried modified FN-GQDs powder was taken, and its mass m_t_ was recorded. Conductometric titration was performed following the same procedure as described above, and the endpoint volume V_t_ was recorded. The carboxyl content after modification and the grafting ratio were calculated using the following formulas:

(2) $${\left[\mathrm{COOH}\right]}_{t}\;\left(\mathrm{mmol}/g\right)\;=\;\frac{C_{\mathrm{NaOH}}\;\times {\;V}_{t}\;\left(\mathrm{mL}\right)}{m_{t}\;\left(g\right)}$$

Formula: $C_{\mathrm{NaOH}}$—concentration, mol/L; $V_{t}$—endpoint volume after modification, mL; $m_{t}$—mass of the modified powder, g.

The grafting ratio of amidine groups was calculated by determining the concentration of carboxyl groups before and after modification using the following formula:

(3) $$\mathrm{Grafting}\;\mathrm{ratio}\;\left(\%\right)\;=\;\frac{{\left[\mathrm{COOH}\right]}_{0}\;-\;{\left[\mathrm{COOH}\right]}_{t}}{{\left[\mathrm{COOH}\right]}_{0}}\;\times \;100\%$$

(3) The viscosity measurement was performed as follows. The constant-temperature water bath was set to 102 °C. Then, 20 mL of the FN-GQDs solution without CO_2_ introduction was transferred into a constant-temperature measuring cylinder, and a No. 0 rotor was used to measure the solution viscosity. CO_2_ was then introduced into the FN-GQDs solution at a constant pressure of 0.3 MPa for 30 min. After 30 min, the viscosity of the CO_2_-stimulated FN-GQDs solution was measured following the same procedure.

(4) The anti-swelling performance test was conducted as follows. First, FN-GQDs solutions of various concentrations were prepared. Then, 0.5 g of bentonite was weighed and placed into a 10 mL centrifuge tube. Then, FN-GQDs solutions of different concentrations were sequentially added to the centrifuge tubes. After shaking to evenly disperse the bentonite, the tubes were allowed to stand for 2 h. Subsequently, the samples were centrifuged at 1500 r/min for 15 min, and the volume of the bentonite was measured. The anti-swelling rate was calculated based on the volume difference using the following formula:

(4) $$B\;=\;\frac{V_{2}-V_{1}}{V_{2}-V_{0}}\;\times \;100\%$$

Formula: B—anti-swelling rate, %; $V_{0}$—swelling volume of bentonite in kerosene, mL; $V_{1}$—swelling volume of bentonite in FN-GQDs solution mL; $V_{2}$—swelling volume of bentonite in water, mL.

**Figure 12:**
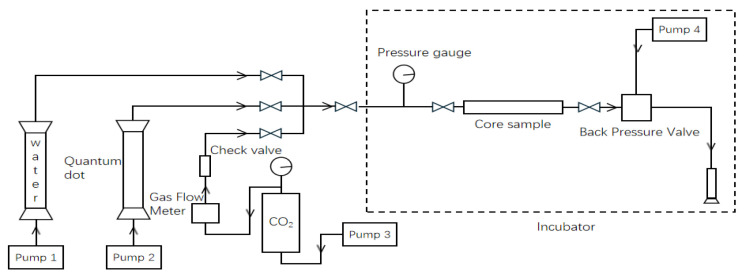
Schematic diagram of the oil displacement experimental apparatus.

(5) The interfacial tension measurement was performed as follows. The oil–water interfacial tension of the carbon quantum dots was determined under experimental conditions at temperatures of 80, 90, 100, 110, and 120 °C, at atmospheric pressure (0.101 MPa). The carbon quantum dots (FN-GQDs) were prepared at a stock solution concentration of 0.02 wt%. A spinning drop high-temperature interfacial tensiometer was used to measure the oil–water interfacial tension at different temperatures.

(6) The wettability reversal test was conducted as follows. Natural cores from the Songliao Basin were used as the experimental physical model, with a gas permeability of 0.2 × 10^−3^ μm^2^. The cores were separately immersed in the 0.02 wt% FN-GQDs solutions at 25 °C, which were purged with CO_2_ and without CO_2_ respectively. CO_2_ was then introduced into the FN-GQDs solution at a constant pressure of 0.3 MPa for 30 min. The contact angle was measured as the water-phase contact angle. A three-phase contact method was employed, using a contact angle goniometer to inject the oil phase beneath the core by means of reverse injection with a syringe, and the variation in the contact angle was monitored. When the contact angle between the core and the oil phase, as well as the immersion solution, was less than 90°, it indicated that the oil phase could not spread on the core surface, suggesting that the core was hydrophilic. Conversely, if the contact angle exceeded 90°, the core was considered oil-wet.

(7) The oil recovery test was conducted as follows. The experimental apparatus used in this study is shown in Figure 12. The oil used in the experiment was live crude oil, prepared by combining reservoir crude oil with dissolved gas in the reservoir, with a viscosity of 0.121 mPa·s at 102.2 °C. First, according to the conventional one-dimensional physical simulation method, the core was sequentially saturated with water and oil. Then, a single-tube displacement model device was connected, and displacement was performed until the water cut reached 95%, during which the oil production from the natural core was recorded. The displacement was carried out at a reservoir temperature of 102.2 °C with a displacement rate of 0.1 mL/min. The displacement methods included CO_2_ flooding and gas–water alternating flooding. For gas–water alternating flooding, 1.2 PV was set as one cycle, and different gas–water ratios were used to control the proportion of gas and water PV within each cycle (PV represents the unit pore volume of the rock). The relationship between cumulative oil production and injection volume was recorded, and the oil recovery curve was plotted. The experimental setup is shown in the figure below.

(8) The core T_2_ spectrum measurement was conducted as follows. A vacuum pump was connected to the NMR core holder, and the core was evacuated for 8 h to remove gas from the core. Subsequently, a prepared deuterium oxide (D_2_O) solution was injected into the core, and the pressure was increased to 36 MPa and remained constant for 72 h (the maximum operating pressure of the NMR system was 40 MPa, with a confining pressure set to 60 MPa and an injection pressure of 37 MPa) to ensure complete saturation of the core with the D_2_O solution. After the core was fully saturated with the D_2_O solution, the internal pressure of the holder was released. The sample preparation device was then connected to the NMR core holder, and the D_2_O solution in the core was displaced with formulated live oil under a formation pressure of 35.7 MPa. Continuous T_2_ spectrum scanning was performed until no further change in the NMR signal was observed, establishing the T_2_ spectrum of irreducible water under initial oil-saturated conditions. After displacement of the core, T_2_ spectrum scanning was performed again until the NMR signal showed almost no further decrease, at which point the experiment was terminated, and the T_2_ spectra after different displacement methods were obtained.

The relaxation time T_2_ was converted into a pore radius distribution curve using the pore throat radius obtained from high-speed mercury intrusion porosimetry, according to the following formula:

(5) $$r\;=\;C\;T_{2}$$

Formula: r—pore throat radius, μm; C—conversion factor, μm/ms; T_2_—relaxation time, ms.

## 5. Conclusions

(1) CO_2_-responsive amidine-functionalized graphene quantum dots (FN-GQDs) were successfully synthesized with an optimal grafting ratio of 58%, which closely matches the 60% saturation value predicted by molecular dynamics simulation. The modified quantum dots remained well-dispersed, with a hydrodynamic size of 26.1 nm.
(2) Upon CO_2_ stimulation, the protonation of amidine groups triggered a sharp, concentration-dependent viscosity increase from 0.298 mPa·s to 2.0 mPa·s at 0.02 wt%, driven by the formation of a dynamic crosslinking network through electrostatic attraction and hydrogen bonding. This in situ viscosification effectively improved the mobility ratio and suppressed gas channeling.
(3) The FN-GQDs exhibited a synergistic triple-function mechanism: (i) ultra-low oil–water interfacial tension (0.12 mN/m) and rapid wettability reversal from oil-wet to water-wet within 80 min, enhancing microscopic displacement efficiency; (ii) excellent anti-swelling performance (92%), which protects water-sensitive shale formations; and (iii) in situ viscosity build-up that enlarges macroscopic sweep volume.
(4) Core-flooding experiments demonstrated that CO_2_/FN-GQDs alternating injection at an optimal gas–water ratio of 2:1 achieved a final oil recovery of 52.5%, which is 21.6 percentage points higher than that of pure CO_2_ flooding. NMR T_2_ analysis confirmed that the incremental oil was mainly mobilized from small pores, highlighting the ability of FN-GQDs to penetrate micro-/nano-pores and release residual oil. This integrated material, combining interfacial activity, anti-swelling protection, and CO_2_-responsive mobility control, shows strong potential for field-scale CO_2_ EOR in shale reservoirs.

## Figures and Tables

**Table 1:** Topological structure of graphene quantum dots with different grafting ratios.

Grafting Ratio (%)	Top View	Side View	Grafting Ratio (%)	Top View	Side View
0 per	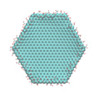	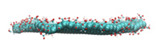	30 per	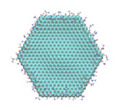	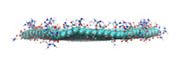
15 per	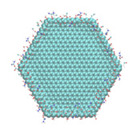	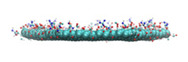	45 per	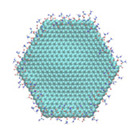	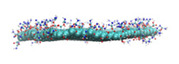
60 per	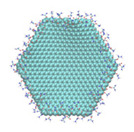	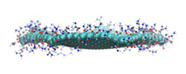	>60 per	structural distortion	

**Table 2:** Composition of simulated formation water.

Sample	KCl	NaCl	Na_2_SO_4_	MgCl_2_·6H_2_O	CaCl_2_	NaHCO_3_
Content/(mg·L^−1^)	8	7581	58	124	96	6120

## Data Availability

All data generated or analyzed during this study are included in this published article.

## References

[1] Lyu W.F., Zhang H.L., Zhou T.Y., Gao M., Zhang D.P., Yang Y.Z., Zhang K., Yu H.W., Ji Z.M., Lyu W.F. (2025). Progress in CO_2_ flooding and storage techniques for lacustrine oil reservoirs and development directions of their large-scale application in China. Pet. Explor. Dev..

[2] Zou C.N., Zhu R.K., Dong D.Z., Wu S. (2022). Technological progress, development strategy, and policy recommendations for shale oil and gas. Acta Pet. Sin..

[3] Ge C.Q., Lei Z.D., Ji D.Q., Zhang Y., Liu Y., Yan Y., Hui G., Chen Z. (2026). Feasibility study on inter-well gas injection for enhanced oil recovery in shale oil horizontal wells. Pet. Sci. Bull..

[4] Pi Z.Y., Hui G., Li J., Yao F., Bao P. (2025). Microscale flow simulation of CO_2_ flooding in shale reservoirs. J. South China Norm. Univ. (Nat. Sci. Ed.).

[5] Song Y., Song Z., Meng Y., Chen Z., Han X., Feng D. (2024). Multi-phase behavior and pore-scale flow in medium-high maturity continental shale reservoirs with Oil, CO_2_, and water. Chem. Eng. J..

[6] Liu F., Gao X., Du J., Lin L., Hou D., Luo J., Zhao J. (2024). Microscopic mechanism of enhancing shale oil recovery through CO_2_ flooding-insights from molecular dynamics simulations. J. Mol. Liq..

[7] Al-Anssari S., Barifcani A., Keshavarz A., Iglauer S. (2018). Impact of nanoparticles on the CO_2_-brine interfacial tension at high pressure and temperature. J. Colloid Interface Sci..

[8] Xia X., Deng Q., Sang P., Tan L., Yan L., Bao J., Li W. (2023). Low-carbon oil exploitation: Carbon dioxide flooding technology. Front. Earth Sci..

[9] Xu C.J., Liu J.X., Xu L., Li M., Luo Q., Pu W.F., Sun L., Dong S., Sun H.Y. (2025). Characteristics of water-alternating-gas assisted CO_2_ flooding in conglomerate reservoirs. Oilfield Chem..

[10] Christensen J.R., Stenby E.H., Skauge A. (2001). Review of WAG field experience. SPE Reserv. Eval. Eng..

[11] Enick R.M., Olsen D., Ammer J., Ammer J.R., Schuller W. (2012). Mobility and conformance control for CO_2_ EOR via thickeners, foams, and gels-a literature review of 40 years of research and pilot tests. Proceedings of the PE Improved Oil Recovery Conference.

[12] Yu K., Zhao K., Ju Y. (2022). A comparative study of the permeability enhancement in coal and clay-rich shale by hydraulic fracturing using nano-CT and SEM image analysis. Appl. Clay Sci..

[13] Zeng F., Zhang Q., Guo J.C., Zeng B., Zhang Y., He S. (2021). Mechanisms of shale hydration and water block removal. Pet. Explor. Dev..

[14] Tang H.M., Gong X.P., Tang H.X., Zhang L.H., Zhao F., He Y. (2016). Evaluation methods and damage mechanisms of shale sensitivity damage. J. Cent. South Univ. (Sci. Technol.).

[15] He J.G., Kang Y.L., You L.J., Cheng Q.J. (2011). Influence of Fluid Damage on Shale Reservoir Stress Sensitivity. Nat. Gas Geosci..

[16] Sheraz M., Wang R. (2025). CO_2_-Responsive Vinyl Polymers: From Synthesis to Application. Molecules.

[17] Li Q., Zhu X., Chen J., Zhao X. (2025). CO_2_ responsive materials in oilfield engineering: Synthesis, mechanisms, and applications. RSC Adv..

[18] Zhao D., Liu Y., Ma Z., Liu J., Wang Y., Wang L., Xia Y., Wang H., Liu Z., Liu X. (2025). CO_2_ adaptive functional materials: Perspectives in geological utilization and sequestration. Adv. Colloid Interface Sci..

[19] Ding M.Y., Jiang Q.Y., Wu P.C., Jin H.B., Yang X.L., Lin S.L. (2025). Research progress of CO_2_-stimuli-responsive polymers. Acta Polym. Sin..

[20] Lu H., Jiang J., Huang Z., Dai S. (2014). A water-soluble CO_2_-triggered viscosity-responsive copolymer of N, N-dimethylaminoethyl methacrylate and acrylamide. J. Appl. Polym. Sci..

[21] Heldebrant D.J., Koech P.K., Ang M.T.C., Liang C., Rainbolt J.E., Yonker C.R., Jessop P.G. (2010). Reversible zwitterionic liquids, the reaction of alkanol guanidines, alkanol amidines, and diamines with CO_2_. Green Chem..

[22] Zhang X.H. (2023). Study on the Performance of CO_2_ Capture by DBN-Diol Solution. Ph.D. Thesis.

[23] Yan Q., Wang J., Yin Y., Yuan J. (2013). Breathing Polymersomes: CO_2_-Tuning Membrane Permeability for Size-Selective Release, Separation, and Reaction. Angew. Chem..

[24] Xiao J., Xiong Y., Dai Y.X., Yang Z.T., Wang X.Z., Zhong L. (2020). Research progress of CO_2_-responsive polymers. Guangzhou Chem. Ind..

[25] Wu G.P., Zhang F.L. (2022). Research progress of nanomaterials for oil displacement. China Surfactant Deterg. Cosmet..

[26] Zhou Y., Shan Y., Xiao L., Song X., Bao W., Ma H., Li H., Wu X., Wang R. (2025). Modified carbon quantum dots based nanocomposite system for enhanced oil recovery in low permeability reservoir. Energy Fuels.

[27] Mohammadi M., Rezvani-Moghaddam A., Roghani-Mamaqani H. (2025). Graphene quantum dots: Effective stabilizing agents for enhanced graphene oxide colloidal dispersions. Colloids Surf. A Physicochem. Eng. Asp..

[28] Bae G., Cho H., Hong B.H. (2024). A review on synthesis, properties, and biomedical applications of graphene quantum dots (GQDs). Nanotechnology.

[29] Medina O.E., Rosales S., Garzon N., López D., Taborda E.A., Ordóñez J.C., Fernández S.A., Cortés F.B., Franco C.A. (2024). Advances in quantum dot applications for the oil and gas industry: Current trends and future directions. Energy Fuels.

[30] Xi F., Zhao J., Shen C., He J., Chen J., Yan Y., Li K., Liu J., Chen P. (2019). Amphiphilic graphene quantum dots as a new class of surfactants. Carbon.

[31] Yin P., Shi F., Luo M., Wu J., Yu Y., Zhang C., Zhao B. (2024). Construction of Carbon Dioxide Responsive Graphene Point Imbibition and Drainage Fluid and Simulation of Imbibition Experiments. Processes.

